# Targeting Notch3 in Hepatocellular Carcinoma: Molecular Mechanisms and Therapeutic Perspectives

**DOI:** 10.3390/ijms18010056

**Published:** 2016-12-28

**Authors:** Catia Giovannini, Luigi Bolondi, Laura Gramantieri

**Affiliations:** 1 Centre for Applied Biomedical Research (CRBA), S. Orsola-Malpighi University Hospital, 40138 Bologna, Italy; luigi.bolondi@unibo.it (L.B.); laura.gramantieri@aosp.bo.it (L.G.); 2 Department of Medical and Surgical Sciences, University of Bologna, 40138 Bologna, Italy

**Keywords:** Notch, p53, hepatocellular carcinoma (HCC)

## Abstract

The Notch signaling pathway is a very conserved system that controls embryonic cell fate decisions and the maintenance of adult stem cells through cell to cell communication. Accumulating evidence support the relevance of Notch signaling in different human diseases and it is one of the most commonly activated signaling pathways in cancer. This review focuses mainly on the role of Notch3 signaling in hepatocellular carcinoma and its potential therapeutic applications against this malignancy. In this regard, the crosstalk between Notch and p53 may play an important role.

## 1. Notch Signaling

The Notch signaling system is crucial for normal development and cell specification in many organs, and it is highly evolutionarily conserved. Four Notch receptors (Notch1, Notch2, Notch3, and Notch4) have been identified in humans and are essential for normal development, with mutations being responsible for CADASIL (Cerebral Autosomal Dominant Arteriopathy with Subcortical Infarcts and Leukoencephalopathy) and Alagille syndromes [1,2]. Notch receptors are heterodimers of two subunits comprising an extracellular domain, rich in EGF repeats, a transmembrane domain and an intracellular domain. The subunits are held together by non-covalent interactions [3].

Notch receptors transduce signals by interacting with transmembrane ligands of the Delta-like (DLL1, 3, and 4) and Jagged (Jagged1 and 2) protein family on neighboring cells [4]. Activating interactions between the receptors and the ligands depend on the interaction of the EGF (Epidermal Growth Factor)-like repeats of the Notch receptors with the DSL domain of the ligands [5]. Ligand binding initiates a series of cleavage events. The first one results in the shedding of the extracellular domain and exposure of a metalloprotease site (S2) which becomes susceptible by the proteases ADAM10 or by TACE (Tumour necrosis factor (TNF)-α converting enzyme). Following this cleavage the resulting Notch fragment, called NEXT (Notch extracellular truncation), is cleaved by γ-secretase and NICD (Notch intracellular domain) is released in the cytoplasm [6]. The activation of Notch largely depends on γ-secretase activity and GSIs (Gamma Secretase Inhibitors) are under evaluation in clinical trials in several human diseases [7,8].

**Figure 1:**
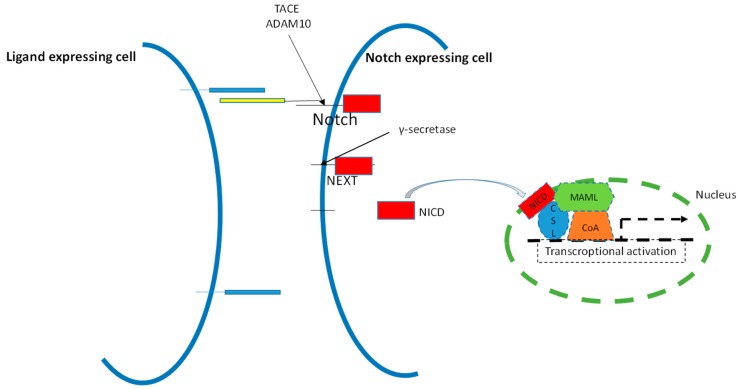
Canonical Notch signaling pathway. Notch receptors are heterodimeric cell membrane proteins containing a 200-kDa extracellular subunit and a 120-kDa fragment that includes a transmembrane and an intracellular domain. Notch receptors are activated by trans-membrane ligands expressed on the surface of neighboring cells. Upon ligand binding, two proteolytic cleavages occur. The first cleavage takes place 12 amino acids outside the trans-membrane domain by metalloproteinase TACE/ADAM10. The resultant Notch COOH-terminal fragment, called NEXT (Notch extracellular truncation), is required for the second cleavage performed by γ-secretase within the trans-membrane region. This last proteolytical event releases a 65–95 kDa Notch intracellular domain (NICD) from the 120-kDa fragment. NICD molecular weight is highly dependent on the Notch receptor that is cleaved. NICD translocates to the nucleus, interacts with DNA binding proteins and transactivates target genes.

NICD is then translocated into the nucleus where it forms a transcriptional complex with the DNA binding protein CSL (CBF1 Suppressor of Hairless Lag1; also called RBP-Jk), Mastermind (Maml), and transcriptional co-activators to drive the expression of Notch target genes (Figure 1). The HES (hairy/enhancer of split) and HERP (HES-related repressor protein) families of transcriptional repressors are the most known targets of Notch signaling [9]. Despite the relative simplicity of the Notch signaling pathway it can generate diverse signaling outputs, as evidenced by profoundly distinct transcriptomic responses in different cell types. Moreover, the inhibition of different Notch receptors has different effects in the liver, underscoring their distinct roles [10,11].

## 2. Notch Pathway in Cancer

During normal development, several mechanisms are used to regulate Notch activity, the best studied of these being lateral contact with neighboring cells, required for correct cell positioning, cell fate, and survival [12]. The Notch family of transcription factors plays critical roles for the efficiency of these fundamental cellular processes in a wide range of tissues. Thus, it is no surprise that the aberrant gain or loss of Notch signaling components has been associated with multiple human disorders including cancer [13,14]. Perturbations of Notch signaling pathway may be caused by an altered expression of Notch receptors or by deregulated expression of Notch ligands. Herein, we focused on Notch receptors whose dysregulation has been found in a variety of human malignancies where they are involved in either survival or death pathways, growth arrest, or proliferation and differentiation [15] (Table 1). The cellular outcome of this aberrant Notch signaling is highly dependent on the context, on the interactions with the tumor microenvironment and crosstalk with other signaling pathways.

## 3. Notch as an Oncoprotein

The first reported Notch1 alteration was the identification of a chromosome translocation by fusing T-cell receptor-β to ICN1 in T-cell acute lymphoblastic leukemia (T-ALL) resulting in constitutive active Notch1 [37]. Then, Aster and Weng reported activated Notch1 mutations in more than 50% of pediatric patients with T-ALL [16,37,38,39,40]. Other studies assessed the prognostic implication of Notch in other leukemia subtypes [22]. Notch1 and Notch2 fusion transcript, retaining the exons that encode the Notch intracellular domain (NICD,) was subsequently described in breast cancer [41,42]. Breast cancer was the first solid tumor in which Notch derangement was molecularly implicated. In line with this evidence, both Notch1 and Notch4 are involved in driving mammary tumorigenesis in mice [17,18]. Moreover, Notch2 may promote the development of estrogen receptor (ER)+ luminal tumors [20]. A role of Notch2 and Akt in breast cancer progression was also supposed [21]. An oncogenic role of Notch2 was described in salivary adenoid cystic carcinoma [36], in bladder cancer [34], and in gastric cancer [35]. Induction of Notch signaling was observed in primary human non-small cell lung cancer (NSCLC) [27]. Notch1 down-regulation inhibits invasion and proliferation of pancreatic cancer cells [29,43]. Notch signaling is overexpressed or constitutively activated in colorectal cancer (CRC) in part because of mutations in regulators of Notch signaling, including *FBXW7* [25,44,45]. More specifically, FBXW7 (SEL-10) functions as an E3 ligase. It binds to the intracellular region of Notch and promotes its ubiquitination and degradation [46,47,48]. Notch3 signaling plays an important role in the proliferation of ErbB2-negative breast tumor cells and its targeted suppression may be a promising strategy for this cancer treatment [19]. Notch3 appears to be a key player in NSCLC, indeed, its suppression results in loss of the malignant phenotype both in vitro and in vivo models [28]. Notch3 amplification was observed in ovarian cancer and is required for tumor proliferation [30]. Aberrant Notch signaling has been found in many other cancers [15,49].

## 4. Notch as a Tumor Suppressor

In some cancers, such as skin squamous cell carcinoma, Notch signaling promotes differentiation and growth arrest [23]. Recently Notch1 mutations have been associated with favorable long-term prognosis in pediatric T-ALL [50]. Giachino et al. identified a tumor suppression function for Notch in forebrain tumor subtype [24]. It has also been reported that Notch may function as tumor suppressor in squamous cell carcinomas (SCCs) of cutaneous, lung, head and neck and esophageal where Notch1 mutations are associated with Notch1 loss of function [26]. The mechanisms by which the Notch pathway can exert tumor promoting or suppressive activity in different cell types are not completely understood and are probably dependent on the microenvironment [51]. It is conceivable that the role of Notch receptors in cancer parallels the role of Notch in tissue development. There are distinct modes of aberrant regulation of the Notch pathway including epigenetic regulation, posttranslational modification, and deregulated expression of Notch ligands [52,53,54,55]. However, the lack of widespread mutational activation in solid tumors leaves doubts about the oncogenic role of Notch [56].

## 5. Notch in Human Hepatocellular Carcinoma

Hepatocellular carcinoma (HCC) accounts for 80%–90% of liver cancers and is the third leading cause of cancer mortality worldwide moreover, HCC is increasing throughout the world [57]. The primary risk factor for HCC is represented by liver cirrhosis, as it is present in the majority of HCC patients [58,59]. Although the majority of cirrhotic patients have a history of viral hepatitis [58,60], metabolic disorders are emerging as etiologic factors for HCC too. Roughly two-thirds of total cirrhotic patients with a viral background are attributed to hepatitis B viral (HBV) infections, while the remaining third are affected by hepatitis C HCV) [59,61]. There is compelling evidence that the Notch signaling pathway may play an important role in HCC. Most of the studies have focused on Notch1, whose role is still ambiguous in terms of anti-tumoral effects following its inhibition [62]. Notch1 increased during the hepatocarcinogenesis process induced by the methyl-deficient diet in the rat liver [63]. Moreover, Notch1 signaling promotes liver carcinogenesis in animal models [31,64]. On the other hand, Huntzicker and coauthors described how Notch1 inhibition altered the proportion of tumor types, reducing HCC-like tumors and increasing cholangiocarcinoma-like tumors [11]. Understanding the control of stem cell proliferation may help us understand the molecular events sustaining hepatocarcinogenesis [65], raising the possibility that Notch1 activity is highly context-dependent. Regarding Notch2 and Notch4, there are studies supporting their role in tumor aggressiveness and metastasis [66,67,68,69]. Specifically, Notch2 signaling was associated with aggressiveness in HCC [66], and its oncogenic potential in the liver was demonstrated in mice whether constitutive Notch2 expression contributes to HCC formation or not [70]. Notch2 inhibition represents a therapeutic option in the treatment of liver cancer [11]. Both Notch2 and Notch4 receptors were found to be involved in the proliferation of hepatoblasts [71]. Notch3 is the only Notch receptor expressed in liver tissue during the middle embryonic stage. Notch3 expression is stronger in the differentiation of stem/progenitor cells (FLSPCs) compared with the adult liver and with the mature rat liver cell line BRL (Buffalo rat liver), respectively. More specifically, as the differentiation of FLSPCs progresses towards hepatocytes, Notch3 protein expression is gradually downregulated, together with the downregulation of other stem cell markers [72]. Conversely, Notch3 results are crucial for bile duct development [73], and its expression persists on the bile duct epithelium in liver tissue [74]. These findings suggest that Notch3 is a potential marker of FLSPCs and regulates the differentiation of FLSPCs into hepatocytes; thus, Notch3 might deserve attention as a possible target driving the use of FLSPCs in the treatment of ESLD (end-stage liver disease). The apparent role of Notch3 in differentiation raises speculations that it might be involved in HCC development. In line with these observations, we reported Notch3 abnormal accumulation in 78% of early HCCs [69]. Bile ducts surrounding HCC nodules stained positive for Notch3 in nearly all cases, raising the question as to whether Notch inhibition might contribute to cholestasis.

Venous endothelia inside the HCC nodule and the stromal cells surrounding neoplastic nodules were positive, while arteriosus endothelia in liver tissue adjacent to HCC nodules were negative. In chronic hepatitis and cirrhosis, Notch3 expression localized to the small- and medium-sized bile ducts [69]. Although functional implications cannot be proven from a descriptive study, the results suggest a possible role of Notch3 in HCC development. In line with these observations, it was reported that Notch3 gene is the most highly upregulated Notch pathway member in HCC tissues compared with normal liver tissue [75]. Although a study conducted in Asiatic patients showed no difference in Notch3 expression between hepatocellular carcinoma and adjacent non-tumor liver cells [76], suggesting that environmental factors are also involved in HCC development, Notch3 should be considered a specific therapeutic target for HCC. Moreover, it is becoming increasingly clear that the effect of signaling proteins is determined not only by the biochemical properties but also by the cellular context in which the protein operates. This also applies to Notch signaling. It is conceivable that both the tumor-suppressive and the oncogenic properties of Notch3 take place at the same time, and the outcome relies upon the cellular context, as described below.

## 6. Notch3 as a Mediator of Chemotherapeutic Resistance in HCC

There is mounting evidence that the Notch pathway contributes to chemoresistance in different human cancers. A recent study on ovarian cancer demonstrated that Notch3 increased resistance to platinum-based chemotherapy and the cancer stem cell population within the tumor [77]. A comparable study in prostate cancer demonstrated that knocking down Notch1 sensitized the cells to treatment with docetaxel [33]. Additional work in glioma reported that Notch promotes both chemoresistance and radioresistance [78]. In HCC models, Notch3 ablation exacerbates the apoptotic response to doxorubicin, which is mostly dependent on p53. [79]. Moreover, we demonstrated that Notch3 inhibition enhances the effect of sorafenib in HCC, in vitro and in vivo [32]. Importantly the effects of Notch3 depletion in sorafenib response are mediated by p21 and pGSK3β^Ser9^ and, probably, by neo-angiogenesis inhibition [32]. Notch3 participates in the process of HCC development in rats treated with diethylinitrosamine, suggesting an active role of Notch3 during carcinogenesis. This observation implies that pharmacological inhibition of Notch3 might reduce HCC development and recurrence after treatments, providing a strong basis for the use of Notch3 inhibition in combination with traditional therapeutic options for the treatment of HCC. γ-secretase inhibitors (GSI) are today evaluated as promising inhibitors of Notch signaling in neoplastic disease [80,81]. RO49229097 is a GSI that showed decreased NICD expression in vitro and in vivo and a significantly inhibition of tumor growth [82]. PF-03084014 is a GSI that has been tested in combination with different treatments in breast, pancreatic, and colorectal cancer [83,84,85], and clinical trials are still ongoing. Unfortunately GSIs are not highly specific [86], have a marked effect on lymphocyte development, and induce changes in gastrointestinal tract [87]. The selective targeting of Notch3 should be preferred for HCC therapy to avoid additional unknown effects to non-neoplastic cirrhotic liver cells that stain negative for Notch3 and positive for others Notch receptors such as Notch4 [69]. Moreover, Pan-Notch inhibitors for HCC could result in stimulating Notch pro-survival activity if not combined with conventional treatment. Indeed, it has been shown that Notch3 inhibition induces a senescent state in HCC cells that depends on p57 accumulation [88].

Senescence inhibits the growth of many tumors including epithelial tumors of the colon, head and neck, and thyroid [89,90,91]. On the other hand, senescent cells in the tumor bulk are important biological factors that might have prognostic implications for the disease outcome. Senescent tumor cells might secrete factors with mitogenic, antiapoptotic, and pro-angiogenic activities [92] leading to a worse prognosis in HCC. In line with the latter, higher p57 protein levels were associated with reduced time to recurrence (TTR) after curative resection [88]. The above considerations recommend caution in suggesting treatments with single general inhibitors of Notch activity (such as γ-secretase inhibitors) for HCC. Approaches that are more promising include selective inhibitors that can suppress the Notch3 pro-survival functions. Alternatively, a combination therapy based on Notch3 inhibitors in conjunction with chemotherapeutic compounds with pro-apoptotic activity should be considered, as we previously showed [32,79]. Moreover, a combination of Notch3 inhibitors with standard therapy might be a good strategy in order to reduce the dosages of the therapeutic agents administrated locally or systemically, limiting side effects and toxicity.

## 7. Notch Crosstalk

**Figure 2:**
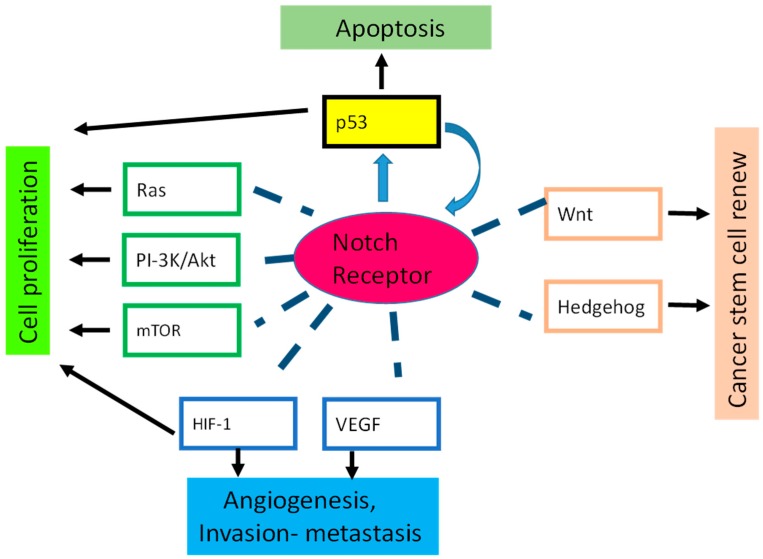
Notch crosstalk. The figure shows the major cellular pathways that interact with Notch signaling. VEGF: vascular endothelial growth factors.

Increasing evidence demonstrates that Notch cross-talks with several conserved pathways mainly involved in stem-cell self-renewal, differentiation, and tumorigenesis (Figure 2). It is also believed that the crosstalk between Notch and other signaling pathways plays an important role in tumor aggressiveness. The manner in which the Notch signals crosstalk with the signals from other pathways is one potential way to affect Notch activity and may be context-dependent.

Notch crosstalk with several conserved pathways including Hedgehog [93,94], Wnt and vascular endothelial growth factors (VEGF) [95,96].

Hypoxia also maintains a stem-cell-like phenotype in colorectal cancer cells in a Notch-dependent manner [97]. Notch cross-talks with the PI3K pathway in breast cancer and is a positive regulator of the PI3K/AKT/mTOR pathway in T-ALL [98,99]. Notch signaling can interface with a large number of cancer-relevant pathways, thus Notch-pathway inhibitors will show optimal efficacy in combination regimens. In this context, Notch interaction with p53 in cancer deserves to be the subject of intensive study [100].

## 8. p53 Is Regulated by Notch in Human Cancer

The tumor suppressor protein p53 acts primarily as a transcription factor, inducing the expression of genes involved in different cellular functions [101]. The efficiency of p53 as a tumor suppressor reflects the alteration of the normal p53 pathway in most cancers, frequently reached by p53 mutations or deletions [102]. However, many other mechanisms involved in p53 regulation, including Notch signaling, have been described. In a mouse model of lymphomagenesis, Notch1 activation reduces p53 levels through increased MDM2 activity [103]. The Notch1/miR-151-5p axis contributed to the progression of gastric cancer cells through downregulation of p53 [104]. NICD-1 inhibits p53 by preventing its phosphorylations at Ser15, Ser20, and Ser392, as well as nuclear localization [105].

In hepatocellular carcinoma, Notch3 regulates p53 at a post-transcriptional level, controlling Cyclin G1 expression and the feed-forward circuit involving p53, miR-221, and MDM2 [106]. More in detail, the increase in p53 protein expression in Notch3-depleted cells is first mediated by a dramatic decrease of Cyclin G1. Then, p53 accumulation in Notch3-silenced cells triggers the upregulation of its known transcriptional target miR-221, leading to MDM2 reduction and thus to increased p53 stability. On the contrary, Notch1 activation upregulates p53 protein expression in HCC cells by inhibiting proteasome degradation [107].

RBP-Jkappa (CSL), an essential mediator of the Notch receptor signaling pathway, binds to the p53 promoter and represses p53 gene expression [108].

A study by Colaluca et al. shows that Numb, which is commonly associated with negative regulation of Notch proteins, plays an important role in the activation of p53 in breast cancer [109]. Loss of Numb is a critical step in the development of breast tumors, leading to the activation of Notch signaling and the inhibition of the p53 pathway [110].

**Figure 3:**
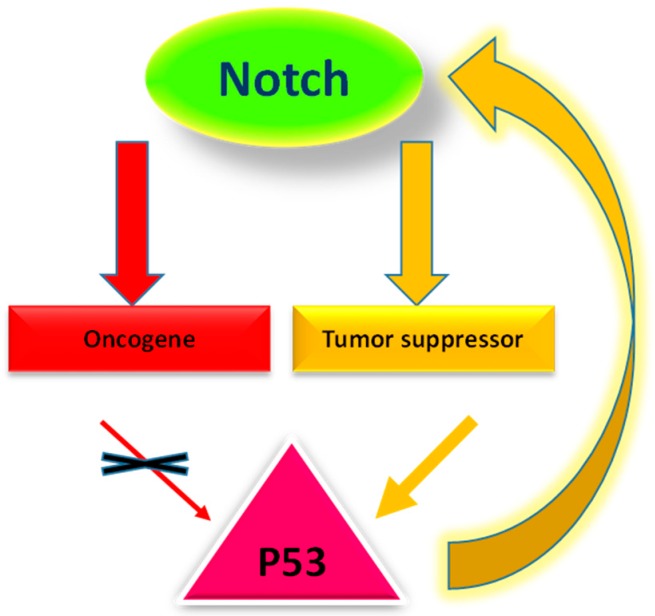
Crosstalk of Notch with p53. Notch acts as an oncogene in cells where it suppresses p53 activity whereas Notch acts as a tumor suppressor in cellular contexts where it supports p53 activation. p53 in turn regulates Notch expression in those tumors in which Notch exerts tumor suppressor activity (yellow representation).

It seems that Notch acts as a tumor suppressor in cells where Notch signaling supports p53 activation. In contrast, Notch frequently acts as an oncoprotein in cells where it suppresses p53 activity (Figure 3). Interestingly, in those tumors in which Notch signaling causes growth inhibition, p53 regulates Notch gene expression and not vice versa [111]. Indeed, p53 was found to specifically regulate Notch1 expression in cervical carcinoma cells [112]. Similar regulation of Notch1 expression by p53 occurs in lung and prostate cancer cells, where increased Notch signaling causes growth inhibition [112]. A probable p53 DNA-binding site about 3.7 kb upstream in the promoter region of the human Notch1 gene was identified [113]. Remarkably, p53 differentially regulated the expression of Notch family members: expression of Notch2 and Notch4 was not induced by p53.

## 9. Conclusions

The Notch signaling pathway is emerging more and more as a major player in cancer biology, including in hepatocellular carcinoma, where persistent Notch activation may lead to HCC proliferation, invasion, and resistance to conventional cancer therapy. In particular, Notch3 seems to be a relevant player in this neoplasia, and its specific inhibition may potentially be more effective than γ-secretase inhibitors that are not cell-selective and possess a considerable toxicity profile. To develop an effective therapeutic approach for HCC treatment, additional studies are required, also taking into account the important regulation mediated by Notch3 on p53.

## Figures and Tables

**Table 1:** Oncogenic and tumor-suppressive role of notch signaling in human cancers.

Tumor Type	Function	Notch Receptor Involved	References
T-cell acute lymphoblastic leukemia (T-ALL)	Oncogenic	Notch1	Weng et al. 2004 [16]
Breast Cancer	Oncogenic	Notch1; Notch4; Notch3; Notch2	Klinakis et al. 2006 [17]; Gallhan et al. 1997 [18]; Yamaguchi et al. 2008 [19]; Fu et al 2010 [20]; Florena et al. 2007 [21]
Acute myeloid leukemia (AML)	Oncogenic	Notch1	Xu et al. 2011 [22]
Tumor of the skin	Tumor-Suppressor	Notch1	Nicolas et al. 2003 [23]
Forebrain Tumor	Tumor-Suppressor	Notch1 and Notch2	Giachino et al. 2015 [24]
Colorectal Cancer (CRC)	Oncogenic	Notch1	Babaei-Jadidi et al. 2011 [25]
Squamous cell carcinomas	Tumor-Suppressor	Notch1	Zhang et al. 2016 [26]
Human non-small cell lung cancer (NSCLC)	Oncogenic	Notch1; Notch3	Westhoff et al. 2009 [27]; Konishi et al 2010 [28]
Pancreatic Cancer	Oncogenic	Notch1	Wang et al 2006 [29]
Ovarian Cancer	Oncogenic	Notch3	Park et al. 2006 [30]
Hepatocellular Carcinoma	Oncogenic	Notch1; Notch3; Notch2	Giovannini et al. 2016 [31]; Giovannini et al 2013 [32]
Cholangiocarcinoma; Prostate Cancer	Oncogenic	Notch1	Huntzicker et al. 2015 [11]; Ye et al. 2012 [33]
Bladder Cancer	Oncogenic	Notch2	Hayashi et al. 2016 [34]
Gastric Cancer	Oncogenic	Notch2	Tseng et al. 2012 [35]
Salivary Adenoid Cystic Carcinoma	Oncogenic	Notch2	Qu et al. 2016 [36]
